# Change in Pediatric Psychiatric Emergency Service Clinicians’ Confidence After Training to Improve Care for Autistic Youth At-Risk for Suicide: A Pilot Study

**DOI:** 10.1007/s10882-025-10025-9

**Published:** 2025-06-18

**Authors:** Paige E. Cervantes, Dana E. M. Seag, Argelinda Baroni, Sarah M. Horwitz

**Affiliations:** 1 Department of Psychiatry, Virginia Commonwealth University, 1308 Sherwood Ave, Richmond, VA USA; 2 Department of Child and Adolescent Psychiatry, NYU Grossman School of Medicine, New York, NY USA; 3 Child and Adolescent Psychiatry, Bellevue Hospital Center, New York, NY USA

**Keywords:** Autism spectrum disorder, Youth suicide, Suicide screening, Emergency psychiatry, Clinician education

## Abstract

Autistic youth visit the emergency department (ED) for psychiatric concerns, including suicidal ideation and behavior, at elevated rates. However, clinicians often report low levels of training and confidence in addressing suicide risk in autistic youth. In this pilot study, clinicians in a pediatric psychiatric emergency service were trained on community- and evidence-informed recommendations to improve suicide-related care for autistic youth and provided with resources to use with autistic youth for a 3-month period. Ratings on attitudes and confidence were obtained from ten providers before and after the training/implementation period and compared. Ratings of feasibility and utility of strategies and resources were obtained from 15 providers after training/implementation and analyzed. While no changes were found across attitudes items, confidence scores were significantly higher after the training/implementation period than before, particularly in the area of suicide risk assessment. Feasibility and utility ratings were generally high, with endorsement patterns aligning with common organizational and systems-level barriers. This pilot study demonstrated that targeted training and evidence-informed recommendations to improve suicide-related care for autistic youth were associated with increased clinician confidence. As research continues in the development of adapted suicide risk assessment tools and management strategies for autistic youth, it is important that both clinical guidance on best practices is provided and that systems-level barriers are addressed.

Although children and adolescents on the autism spectrum present to emergency department (ED) settings at elevated rates, EDs are not designed to accommodate these youth, and barriers to optimal care are numerous. ED providers’ knowledge related to autism is often limited, and accommodations are rarely provided. The ED environment is also challenging, as exposure to aversive sensory stimuli, including but not limited to loud noises, crowded waiting rooms, and touch during evaluation, is frequently unavoidable. Further, autistic youth often have difficulties coping with the long wait times, frequent transitions, and interactions with multiple care staff within an ED visit (Muskat et al., 2016; Nicholas et al., 2016; Sadatsafavi et al., 2023; Zwaigenbaum et al., 2016).

Further complicating ED care, autistic youth are nine times more likely to present to the ED with psychiatric concerns (Kalb et al., 2012). In fact, recent research demonstrated that pediatric ED visits for suicidal ideation and behavior were significantly more prevalent and have increased significantly more over time in autistic compared to non-autistic patients (Cervantes, Brown et al., 2023). This is problematic, as ED providers often report little training and low confidence in assessing and managing mental health symptoms and suicide risk in youth broadly (Betz et al., 2013; Cervantes et al., 2023b; Habis et al., 2007; Rudloff et al., 2021). As could be expected, clinicians endorse significantly more limited training and confidence when addressing suicide risk in autistic youth specifically (Cervantes, Li, et al., 2023; Cervantes et al., 2025). A recent qualitative study found that autistic youth, their caregivers, and ED clinicians reported several challenges related to suicide-related care in emergency settings, with the most widely endorsed being limited workforce capacity. Among other concerns were difficulties managing the language demands and the reliance on emotion recognition and expression within psychiatric evaluation and suicide risk management strategies, difficulties coping with the nature of ED visits and the sensory environment, particularly when in psychiatric distress, and concerns about provider approach to integrating caregivers into the assessment and treatment process. Service system barriers were frequently reported and related to the accessibility, availability, and affordability of specialized services (Cervantes et al. 2024).

Advancements in psychiatric care in the ED for youth on the autism spectrum are urgently needed. EDs are often recognized as a critical point for suicide prevention (Brahmbhatt et al., 2019). However, despite the prevalence of suicidal thoughts and behaviors and the incidence of suicide death being significantly higher in the autism population than in the general population (Kõlves et al., 2021; Segers & Rawana, 2014), current recommendations related to suicide prevention practices in the ED do not offer guidance specific to autistic youth. Further, there are currently no suicide risk screening tools validated for use with autistic children and adolescents, and gold standard suicide risk management practices have not been formally evaluated in this group. It is highly likely that adaptations to these practices are necessary to improve efficacy and acceptability (Schwartzman et al., 2021).

In the absence of evidence-based strategies specific to the autism population, ED clinicians require training on how to improve care for autistic youth in psychiatric crisis, with a focus on suicide risk assessment and management practices. Therefore, this pilot study aimed to educate and provide evidence-informed resources to support clinicians in a pediatric psychiatric emergency service to better serve these youth. The effect of the training and resources was evaluated using pre- post-surveys measuring confidence prior to the training and after a 3-month implementation period. Perceived feasibility of the strategies and utility of the provided resources were assessed in a survey following implementation.

## Method

### Participants

Participants were clinicians from a pediatric psychiatric emergency service at a public hospital in New York City. This setting provides comprehensive care for youth presenting to the hospital in psychiatric crisis (Gerson et al., 2017) and is staffed by mental health specialists including child and adolescent psychiatrists, psychologists, and social workers as well as trainees (i.e., psychiatry fellows and psychology interns). While these clinicians have expertise in managing psychiatric crisis and suicidality broadly and they work with autistic patients often, they are not necessarily specialized in autism. In fact, previous research documented that their self-reported training and confidence levels were significantly lower when providing suicide-related care to autistic compared to non-autistic youth (Cervantes, Li, et al., 2023). The autism training and evidence-informed resources described in this study were offered to all clinicians in the pediatric psychiatric ED as part of a quality improvement effort. All clinicians were also invited to complete the associated surveys. Participation in these surveys was voluntary.

### Procedures

This study was conducted as part of an IRB-approved quality improvement effort to offer training in autism and suicide risk and to evaluate a screening program for autistic youth implemented within a larger NIMH-funded initiative to advance mental health screening in EDs for all youth aged 7–17. Within this study, pediatric psychiatric ED clinicians were asked to participate in a new screening program for all patients using a novel tool to assess mental health symptoms, to attend an educational session on autism and suicide risk, and to complete anonymous, online surveys at three time points. To complete the surveys, clinicians were sent an email with a link to the survey on REDCap, a secure web-based data management platform, at each time point. Clinicians were required to indicate informed consent via REDCap prior to accessing the first survey. Recruitment for each survey was open for 4 weeks, and clinicians were sent up to two email reminders about the study during that time. The first survey was administered at baseline, prior to the implementation of the novel screening program for youth broadly. The second survey was administered after the introduction of the screening program but prior to any autism-specific training. The third survey was administered after the autism educational session and the introduction of evidence-informed resources specific to modifying care for autistic youth, with a focus on suicide risk assessment and management.

The autism training was delivered over videoconference for one hour. The session included information on the prevalence of and risk factors for suicidal thoughts and behaviors in autism, the frequency at which youth on the autism spectrum present to the ED and barriers to quality ED care, common barriers to adequate mental health care for autistic youth, and recommendations for modifying care to improve suicide risk assessment and management and autistic patient experience. Information and recommendations included in the training were informed by baseline data in the pediatric psychiatric emergency service (e.g., prevalence of autistic patients seeking care, differences - or lack thereof - in rates of suicide risk across autistic and non-autistic patients), perspectives from the autism community obtained in qualitative interviews (Cervantes et al., 2024), and available research literature. The strategies discussed in the training are presented in Table 1. The training was delivered by a clinical psychologist who specializes in autism and co-occurring mental health challenges. After the training, implementation of recommendations was supported by the provision of evidence-informed resources for approximately 3 months. During this time, when a patient participated in the larger quality improvement initiative and had a confirmed or suspected autism diagnosis, the research team provided clinicians with a recommendation sheet that presented a brief review of strategies from the training, visuals to use during their evaluation to enhance communication, a visual schedule detailing common steps for the pediatric psychiatric ED visit to share with the patient, and a list of outpatient clinics that offer services to autistic youth for referrals. These resources were also posted in clinician offices for use with autistic patients who did not participate in the initiative or who presented during a time when the research team was not available.

**Table 1:** Overview of recommendations presented in training

**Optimizing Communication**
- Take time to develop rapport - Ask direct, clear questions; give context to prevent misinterpretations; avoid nonliteral language - Use fewer words; allow time for processing - Check for mutual understanding by reflecting patient responses back to them for confirmation - To address any anxiety related to providing accurate information, give reminders that it is okay to provide a “best guess” to your questions - Understand emotion recognition and expression may be difficult for some autistic youth and instead focus on more explicit indicators of distress, such as physiological signs and behaviors - When discussion of emotions is needed, provide and define emotion vocabulary - Do not rely on nonverbal expression to estimate level of distress - Use visual supports to support patients in responding to your questions and when introducing a new concept - Offer alternate communication methods (e.g., writing, drawing, typing) - If perseverative, help shift thoughts by discussing preferred topics or engaging in preferred activities before returning to your evaluation - If emotionally dysregulated, pause the evaluation and give youth space
**Developing Expectations**
- Provide a visual schedule presenting the steps and processes involved in the ED visit - Formally introduce team members who will interact with the patient - Clearly explain rationale behind questions/procedures as well as any time constraints - Make uncertainty clear from the start (e.g., other team members who may be involved, admission decision and length to be determined following evaluation)
**Addressing Sensory Environment**
- Ask about sensory experience and needed supports before beginning evaluation - If possible, minimize wait time for autistic patients and their families - Provide distractions in the waiting room (e.g., sensory items, snacks) - Provide care in a private room with few distractions, farther away from loud or crowded areas
**Relevant Suicide Risk Assessment Factors**
- Ask follow-up questions to suicide risk screening responses to ensure accurate interpretation (e.g., “What does that mean to you?”) - Assess presence of warning signs and changes from baseline to differentiate mental health symptoms
**Managing Suicide Risk**
- Safety plan should be concrete and written (e.g., coping strategies, distractions, and contacts should be identified across settings and contexts relevant to the youth) - Incorporate special interests - Identifying and practicing coping strategies and warning sign recognition may require more time and explicit teaching - Involve family members in treatment plan (e.g., to reinforce warning sign recognition and use of safety plan) following discussions about their involvement with the patient - Ask about contact preferences; write down and practice what to say when contacting clinical resources for help
**Developing a Care Plan**
- Explain patient’s full clinical picture (e.g., autism features, mental health symptoms, and how they may interact) - Discuss treatment plan and coordinate with child’s full care team (e.g., caregivers, school, outside providers) - Refer to outpatient mental health clinic that accepts patients with an autism diagnosis - Ask about formal education and developmental disability services; refer for more comprehensive supports - If not formally diagnosed, refer for diagnostic evaluation

### Measure

As described previously (Cervantes, Li, et al., 2023), the clinician survey inquired about the assessment and management of suicide risk. It was administered up to three times during the study. Surveys collected information about demographic and work status (first administration only), training history (first administration only), and attitudes and confidence (all administrations). The attitudes and confidence items were modified from the scale used in Betz and colleagues’ (2015) study of ED providers and were rated on a 5-point Likert scale (i.e., “1” = strongly disagree; “5” = strongly agree). Items related to addressing suicide risk in both the autism population and in the general child and adolescent population were presented but only the autism items were analyzed in the current study. Four items inquired about attitudes toward suicide risk screening and management for autistic youth. Six items pertained to confidence providing suicide-related care to autistic youth and were summed to calculate a total confidence score. At the final administration, we included items to assess utility of the training (yes/no) and provided resources (“1”=not useful; “5”=very useful) as well as the feasibility of recommended strategies (“1”=unfeasible; “5”=very feasible) in serving children and adolescents on the autism spectrum.

### Statistical Analysis

Change in total confidence scores obtained before and after the training/resource implementation period was analyzed using a paired-samples *t*-test. Wilcoxon signed-rank tests were performed to evaluate change in item level endorsements across items assessing attitudes and confidence. Ratings of feasibility and utility obtained after training/implementation were evaluated descriptively.

## Results

Over 90% of all clinicians working in the pediatric psychiatric ED service at the time of the training attended the educational session. Twenty-seven clinicians consented to participate and completed at least one survey. In part because trainees typically rotate through the pediatric psychiatric ED service every few months, fewer were retained for the entirety of the study. Four clinicians completed all three surveys, and six clinicians completed two of the three. Because we did not see or expect to see changes in clinician confidence scores across baseline and the second administration of the survey, we used either baseline (*N* = 4) or the second (*N* = 2) survey data to serve as a pre-training estimate of attitudes and confidence related to suicide risk assessment and management for autistic youth. When a participant had completed both baseline and second surveys (*N* = 4), we averaged their ratings on these surveys to serve as the pre-training scores. In total, ten clinicians were included in the pre- post-analyses. An additional five clinicians completed only the final survey and were included in analyses of feasibility and utility. Therefore, the total sample consisted of 15 clinicians. Two clinicians did not report their demographic information. Of the remaining 13 clinicians, eight were child and adolescent psychiatrists, psychologists, and social workers on staff, and five were trainees (i.e., psychiatry fellows and psychology interns). Eight clinicians had MDs, one had a PhD, three had MSWs, and one had another Master’s degree. Participants primarily identified as White (*N* = 8) and non-Hispanic/Latino (*N* = 11), were female (*N* = 9), and were under the age of 40 (*N* = 11).

There were no statistically significant differences in item endorsements across attitude items before and after the training/implementation period. However, total confidence scores were significantly higher after the training/implementation period (*M* = 24.10; *SD* = 4.28) compared to before (*M* = 21.40; *SD* = 5.32), *t*(9)=-2.31, *p* = 0.046. Upon further investigation of item level differences, significantly higher endorsements were found after training/implementation on provider confidence in their ability to further assess suicide risk in autistic patients, *z*=-2.41, *p* = 0.016. Change in ratings on their ability to screen autistic youth for suicide risk approached statistical significance, *z*=-1.87, *p* = 0.062 (Table 2).

**Table 2:** Change across Pre- and Post-Training/Implementation survey ratings

	Pre-Training/ Implementation			Post-Training/ Implementation			*p*
	M	SD	Med.	M	SD	Med.	
**Attitudes Items**							
I believe addressing the co-occurrence of ASD and suicidality is important.	4.50	0.47	4.5	4.60	0.52	5	ns
I believe adaptations are necessary to standard suicide risk assessment and management practices when caring for youth with ASD.	4.30	0.42	4	4.40	0.52	4	ns
I believe screening for suicide in youth with ASD in the hospital’s EDs is important.	4.20	0.75	4.25	4.50	0.53	4.5	ns
My colleagues believe screening for suicide in youth with ASD in the hospital’s EDs is important.	4.05	0.83	4	4.40	0.52	4	ns
**Confidence Items**							
I have the skills needed to screen patients with ASD for suicidality.	3.50	0.94	4	4.10	0.74	4	0.062
I am confident in my ability to further assess a patient with ASD’s suicide risk severity.	3.50	0.94	4	4.20	0.63	4	0.016
I know how to provide brief counseling to suicidal patients with ASD.	3.50	1.29	3.75	4.00	0.94	4	ns
I am confident in my ability to discuss suicide risk and access to lethal means with parents/guardians of patients with ASD.	4.05	0.50	4	4.30	0.48	4	ns
I am confident in my ability to help patients with ASD at risk of suicide create a personalized safety plan.	3.75	1.09	4	4.10	0.88	4	ns
I am confident in my ability to help find referral resources for suicidal patients with ASD.	3.10	1.02	3	3.40	0.97	3.5	ns
**Total Confidence Score**	**21.40**	**5.32**	**22.75**	**24.10**	**4.28**	**23.5**	**0.046**

All survey participants who reported attending the training indicated that it was useful. Mean feasibility ratings obtained following the training/implementation period regarding strategies presented within the training ranged from 3.53 to 4.20. Recommendations presented on modifying suicide risk assessment (*M* = 4.20; *SD* = 0.77), adapting risk management practices (*M* = 4.14; *SD* = 0.66) and optimizing communication (*M* = 4.07; *SD* = 0.88) all received mean ratings above 4 (i.e., “feasible”). Mean feasibility ratings for strategies related to developing expectations (*M =* 3.93; *SD =* 0.96), developing a care plan after discharge (*M =* 3.73; *SD* = 1.03), and modifying the sensory environment (*M* = 3.53; *SD* = 0.92) were lower but remained neutral. Utility ratings of the resources provided within the implementation period ranged from 3.92 to 4.50. The outpatient referral list had the highest mean rating (*M* = 4.50; *SD* = 0.67), followed by the visual schedule (*M =* 4.00; *SD* = 0.74), visual communication aids (*M* = 3.92; *SD* = 0.86), and recommendation sheet (*M* = 3.92; *SD* = 1.04) (Table 3).

**Table 3:** Feasibility and utility ratings after training/implementation

**Feasibility Ratings for Strategies**	***M (SD)***
Modifying risk assessment	4.20 (0.77)
Adapting risk management	4.14 (0.66)
Optimizing communication	4.07 (0.88)
Developing expectations	3.93 (0.96)
Developing a care plan after discharge	3.73 (1.03)
Modifying the sensory environment	3.53 (0.92)
**Utility Ratings for Resources**	***M (SD)***
Outpatient referral list	4.50 (0.67)
Visual schedule	4.00 (0.74)
Visual communication aids	3.92 (0.86)
Recommendation sheet	3.92 (1.04)

## Discussion

Given the increased suicide risk in youth on the autism spectrum (O’Halloran et al., 2022), particularly in conjunction with higher ED utilization rates for psychiatric concerns (Cervantes, Brown et al., 2023; Kalb et al., 2012), it is imperative that ED providers are competent and confident addressing suicidality in autism. However, clinicians often indicate feeling ill-equipped to care for individuals on the autism spectrum (Cervantes et al., 2025; Cervantes, Li, et al., 2023; Jager-Hyman et al., 2020). In the current pilot study, pediatric psychiatric emergency service providers reported only moderate levels of confidence addressing suicide risk in autistic youth at baseline despite expertise in assessing and managing youth mental health crises in the general population. Encouragingly though, even in this small sample, providing training on suicide risk in autism and evidence-informed resources was associated with a statistically significant increase in provider confidence working with autistic youth, particularly in the area of suicide risk assessment. These findings suggest that while suicide risk assessment tools and management practices are being evaluated and modifications developed for the autistic community over the next several years, evidence- and community-informed strategies to improve care can be disseminated with benefit to provider confidence. This is critical, as there is an urgent need to address the co-occurrence of autism and suicide risk yet limited guidance for clinical considerations available in the literature (Schwartzman et al., 2021).

Clinician ratings on feasibility of recommended strategies were also encouraging. In the context of a high-paced emergency psychiatric setting, even with increased demands due to the COVID-19 pandemic when these data were collected, clinicians considered the strategies presented mostly feasible. Lower mean feasibility ratings were found in domains that appeared more related to organizational (e.g., managing the sensory environment of the ED and unpredictability of the visit) and systems-level (e.g., the availability of outpatient referral options for autistic youth after discharge) barriers than individual-level barriers. Aligned with this, the referral list of outpatient clinics that offer services to autistic youth in the area received the highest mean utility rating across all resources provided. This was not surprising. Previous research had found that securing services for autistic youth with psychiatric concerns is difficult (Cervantes et al., 2024), and a minority of public outpatient clinics in New York City accepted youth on the autism spectrum as patients (Cervantes, Conlon., 2023). Lower mean utility ratings were found for the recommendation sheet and the visual communication aids. In a previous study, ED clinicians discussed potential difficulties employing communication strategies in particular, given the amount of practice required to become skilled at implementing a modified approach to care for autistic youth (Cervantes et al., 2024). Therefore, more intensive training and/or feedback regarding implementation of some strategies may be beneficial in improving the utility of these recommendations and resources as would be ongoing support from autism specialists.

There are limitations to this pilot study. The sample size was small and included a diverse group of mental health professionals employed in a specialized setting. Few pediatric psychiatric ED services exist across the country, and baseline ratings were likely higher because these clinicians were mental health specialists. Autistic youth may be more likely to visit a general ED, perhaps with fewer mental health resources. Therefore, future research should focus on better preparing providers in these general settings to address suicide risk in autism. Given the documented limitations clinicians in pediatric EDs demonstrate in managing youth psychiatric concerns broadly (Betz et al., 2013; Cervantes et al., 2023b; Habis et al., 2007), these efforts may require a more intensive approach. In fact, training and support for the implementation of evidence-informed resources with examination of and feedback on clinician fidelity would likely improve outcomes. Future research should work to identify optimal training methods to improve not only clinician confidence but also clinical care, patient satisfaction, and patient outcomes.

Despite these limitations, this pilot study demonstrated that targeted training in community- and evidence-informed recommendations to improve suicide-related care for autistic youth was associated with increased clinician confidence. Given the urgency of intervening on the long under-recognized increased suicide risk within this population, training initiatives to increase provider competence and confidence should be expanded. This is particularly important in the ED, since it is an essential point of psychiatric care for autistic youth (Kalb et al., 2012). Such efforts should include autistic community perspectives and priorities while optimizing feasibility for clinicians. However, it is important to note that these initiatives cannot succeed without attention to systems-level issues, including lack of mental health service availability and an insufficient autism specialty workforce. Because research has shown that unmet mental health needs are associated with a higher likelihood of ED utilization in the autism population (Badgett et al., 2023), comprehensive and coordinated efforts are essential to address barriers to quality suicide-related care in EDs.
